# Synthesis and Characterization of Cellulose Acetate/Polyethylene Glycol/Poly(Styrene)-b-Poly(4-Vinylpyridine) Membrane Embedded with Hydrotermaly Activated TiO_2_ Nanoparticles for Waste-Waters Treatment by Membrane Processes

**DOI:** 10.3390/polym17040446

**Published:** 2025-02-08

**Authors:** Simona Căprărescu, Grațiela Teodora Tihan, Roxana Gabriela Zgârian, Alexandru Mihai Grumezescu, Carmen Lazau, Cornelia Bandas, Leonard Ionuț Atanase, Cristian-Andi Nicolae

**Affiliations:** 1 Department of Inorganic Chemistry, Physical Chemistry and Electrochemistry, Faculty of Chemical Engineering and Biotechnologies, National University of Science and Technology POLITEHNICA Bucharest, 1-7 Polizu Street, 011061 Bucharest, Romania; simona.caprarescu@upb.ro; 2 Department of General Chemistry, Faculty of Chemical Engineering and Biotechnologies, National University of Science and Technology POLITEHNICA Bucharest, 1-7 Polizu Street, 011061 Bucharest, Romania; gratielatihan@yahoo.com; 3 Department of Science and Engineering of Oxide Materials and Nanomaterials, Faculty of Chemical Engineering and Biotechnologies, National University of Science and Technology POLITEHNICA Bucharest, 1-7 Polizu Street, 011061 Bucharest, Romania; agrumezescu@upb.ro; 4 Research Institute of the University of Bucharest, University of Bucharest, 90 Panduri Street, 050663 Bucharest, Romania; 5 National Institute for Research and Development in Electrochemistry and Condensed Matter Timisoara, Dr. A.P. Podeanu no. 144, 300569 Timisoara, Romania; carmen.lazau@gmail.com (C.L.); cornelia.bandas@gmail.com (C.B.); 6 Faculty of Medicine, Apollonia University of Iasi, 700511 Iasi, Romania; leonard.atanase@yahoo.com; 7 Academy of Romanian Scientists, 050045 Bucharest, Romania; 8 National Institute for Research & Development in Chemistry and Petrochemistry—ICECHIM, 202 Splaiul Independentei, 060021 Bucharest, Romania; ca_nicolae@yahoo.com

**Keywords:** TiO_2_, polymeric membranes, polymers, properties, characterizations

## Abstract

This study investigated the properties of a novel polymeric membrane based on cellulose acetate, polyethylene glycol/poly(styrene)-b-poly(4-vinylpyridine), and embedded with TiO_2_ nanoparticles (CA/PEG/PS_154_-b-P4VP_381_/TiO_2_ membrane) obtained by wet-phase inversion method. The TiO_2_ nanoparticles fabricated by a hydrothermal method were characterized by XRD, SEM, EDX, and UV-Vis analyses to determine the purity, morphology, and optical band gap energy. The prepared polymeric membranes with and without TiO_2_ nanoparticles (CA/PEG/PS_154_-b-P4VP_381_/TiO_2_ and CA/PEG/PS_154_-b-P4VP_381_ membranes) were characterized by FTIR, SEM, EDXS, and TGA to observe the effect of TiO_2_ nanoparticles added to the polymeric membrane matrix and to analyze the chemical structure, morphology, and thermal stability of the obtained polymeric membranes. The contact angle, SFE, water retention, and porosity were also determined. The results showed that adding the TiO_2_ nanoparticles into the polymeric membrane (CA/PEG/PS_154_-b-P4VP_381_/TiO_2_) significantly reduced the pore size and the water contact angle, increasing the water retention and the porosity. The lower value of the water contact angle of 15.57 ± 0.45° for the CA/PEG/PS_154_-b-P4VP_381_/TiO_2_ membrane indicates a pronounced hydrophilic character. The investigations performed showed that the CA/PEG/PS_154_-b-P4VP_381_/TiO_2_ membrane presents excellent properties and can be a promising material for water and waste-water treatment through membrane processes (e.g., electrodialysis, ultrafiltration, nanofiltration, reverse osmosis) in the future.

## 1. Introduction

In recent years, there has been increased interest in the synthesis of polymeric membranes containing inorganic nanoparticles and establishing structure-properties relationships between natural or synthetic polymers and inorganic nanoparticles in the membrane matrix for successful use in different membrane processes (nanofiltration [1,2], ultrafiltration [3,4,5], forward osmosis [6], electrodialysis [7], and others [8,9,10]) to treat or purify various waters and waste-waters. The successful production of a polymeric membrane containing inorganic nanoparticles from small (laboratory) to large (industrial) scales depends on the preparation methods, but also on the relevant conditions and parameters for the synthesis, including types of raw materials, selection of the polymers (chitosan, cellulose acetate, polyvinyl alcohol, poly(ethylene glycol), polysulfone, and derivates, polyethylene, polyvinylidene fluoride, polyetheretherketone, and many more [1,5,7,8,9,10]), optimal amount and concentration of the polymers, selection of the solvent, optimal amount and concentration of the solvent, selection of solvent/non-solvent system, additives properties (e.g., hydrophobicity/hydrophilicity, permeability, ion exchange capacity, amphiphilic surface with fouling-resistance, antifouling, solubility, water retention capability), pore-forming agents, coagulation (mixture of solvents), polymer/solvent ratio, amount and size of nanoparticles, distribution of the nanoparticles in the polymeric solution, sintering temperature, and stirring times.

The most widely used methods or techniques for preparing the polymeric membranes are wet phase inversion, interfacial polymerization, stretching, track etching, sintering, solvent-induced phase separation, vapor-induced phase separation, and electrospinning [11,12,13]. All synthesis methods present advantages and disadvantages [1,3,6,10,11,12,13]. Still, the biggest challenges regarding the fabrication of polymeric membranes containing inorganic nanoparticles applicable in water and waste-water treatment refer to the incompatibility between organic and inorganic phases, which can produce flaws in the polymeric mixture, agglomeration of the inorganic nanoparticles, which can cause the formation of macro pores/macro-voids, and excellent properties (chemical, mechanical, and thermal stability, biodegradability, strength, and fouling resistance, permeability, selectivity, performance, and separation efficiency).

Wet-phase inversion is the most favorable method for the preparation of the polymeric membranes containing inorganic nanoparticles at a laboratory scale due to their advantages. These include easy, simple, fast, and economical processing, control of synthesis parameters (e.g., temperature, duration time, stirring speed), low energy consumption, being environmentally friendly, not requiring expensive equipment or devices, accessibility of raw materials (e.g., polymers, copolymers, solvents, additives), requiring small amounts of the raw materials, and production of small amounts of waste [7,13,14,15,16].

The beneficial effects of including inorganic nanoparticles (zinc oxide (ZnO) [12,14], silica (SiO_2_) [9,12,15], aluminum oxide (Al_2_O_3_) [16,17], zirconium dioxide (ZrO_2_) [8,18], cerium oxide (CeO_2_) [12,19], titanium dioxide (TiO_2_, titania)) [20,21,22,23,24] on the properties of polymeric membranes are known. Căprărescu et al. [7] found that the addition of SiO_2_ into the polymeric matrix containing polyvinyl alcohol, acrylonitrile, and vinyl acetate reduces the macro void formation in the membrane structure, changes the chemical structure, increases the protonic conductivity, and favorably influences the membrane performance in terms of demineralization and metal ion removal rate. Heng et al. [15] reported that the modified polysulfone membranes loaded with carbon dots and silica (SiO_2_) improved the properties of the obtained membrane, such as pore size, hydrophilicity, porosity, and permeability. All the modified membranes exhibited higher water throughput and dye rejection. A higher water permeability was obtained for the membrane that was dopped with 1.0 wt.% of carbon dots and SiO_2_. Yan et al. [17] showed that adding Al_2_O_3_ nanoparticles into a membrane matrix containing dimethylacetamide and polyvinylidene fluoride increased the hydrophilicity of the membrane and water flow permeation. Bottino et al. [18] demonstrated that increasing the amount of ZrO_2_ in the poly(vinylidene) fluoride composite membranes leads to an increase in permeate flux but a decrease in pollutant retention. In another study, Tavangar et al. [19] demonstrated that the incorporation of cerium oxide (CeO_2_) nanoparticles into the poly(ether sulfone) nanocomposite membranes contributed favorably to the membrane properties (hydrophilicity, pore size), the water flux, the contact angle, and the pollutant rejection.

TiO_2_ nanoparticles are widely used in the membranes matrix due to their advantages and properties, including availability, abundance, low cost, non-toxicity, biocompatibility, hydrophilicity, environmental friendliness, chemical and thermal stability [20,21,22,23,24], mechanical resistance, and antibacterial and photocatalytic activity [24,25,26]. Several researchers have fabricated and studied various modified membranes by incorporating different amounts of polymers, additives, and TiO_2_ into the membrane matrix. Wibowo et al. [8] prepared a semi-permeable membrane based on cellulose acetate, polyethylene glycol, and chitosan enriched with TiO_2_ nanoparticles by the blending method. They reported that increasing the amount and concentration of TiO_2_ nanoparticles in the membrane composition affected the strength and permselectivity properties. Ahmad et al. [10] prepared a composite polymeric membrane containing polyurethane/cellulose acetate and functionalized TiO_2_ nanoparticles using the solution casting and solvent evaporation technique. They reported that an increase of TiO_2_ nanoparticles concentration in the polymer solution matrix from 0.5 to 1.5 wt.% resulted in the facilitation of macro-void formation in the membrane structure. Also, adding a higher concentration of TiO_2_ nanoparticles increased the stability and enhanced the hydrophilicity of the membrane, increasing the water flux and retention. Aparicio et al. [22] synthesized polyvinyl alcohol/TiO_2_ polymer membranes cross-linked with glutaraldehyde solution using a solution casting method. They reported that the polymer membranes exhibited good thermal stability, and the ionic conductivity of these membranes was conditioned by swelling degree. Nascimben Santos et al. [23] investigated the operational synthesis conditions and performance of two polymers (polyvinylidene fluoride and polyvinylpyrrolidone) and TiO_2_ membranes. These membranes were obtained by the phase inversion/immersion precipitation method. They demonstrated that the pore size and water flux are not influenced by the amount of TiO_2_ but by the polymer concentration. Shafiq et al. [24] prepared the cellulose acetate/polyethylene glycol composite membranes by adding TiO_2_ nanoparticles using a dissolution casting method. They reported that a higher concentration of TiO_2_ nanoparticles (25 wt.%) increases thermal stability and the permeation flux. Chi et al. [27] demonstrated that the obtained polyamide composite membrane enriched with TiO_2_ nanoparticles enhanced the membrane’s selectivity, porosity, hydrophilicity, and water permeability.

This work proposes to fabricate a novel polymeric membrane based on cellulose acetate, polyethylene glycol/poly(styrene)-b-poly(4-vinylpyridine), embedded with TiO_2_ nanoparticles (CA/PEG/PS_154_-b-P4VP_381_/TiO_2_ membrane) through the wet-phase inversion method. The effects of TiO_2_ nanoparticles into the polymeric membrane and the changes were investigated by FTIR, SEM, EDXS, and TGA. FTIR, SEM, and TGA analyses were used to examine the chemical structure, the surface morphology, and the thermal stability of the fabricated polymeric membranes. Other characterizations and supplementary investigations (water contact angle, SFE, water retention, porosity) of the polymeric membranes were evaluated to determine the optimal conditions under which polymeric membranes with specific properties can be fabricated for further use in water and waste-water treatment by membrane processes (e.g., electrodialysis, ultrafiltration, nanofiltration, reverse osmosis).

## 2. Materials and Methods

### 2.1. Materials and Chemicals

Titanium (IV) chloride (TiCl_4_, 99.9%) and oxalic acid (HO_2_CCO_2_H, 99.0%) of analytical purity grade were purchased from Sigma-Aldrich Company (St. Saint Louis, MI, USA) and were used with no purification. Cellulose acetate (CA) powder, dimethyl sulfoxide (DMSO) (liquid), glycerol (liquid), and polyethylene glycol 400 (PEG) (liquid) (density 1.125 g cm^−3^, average molar mass 400 g mol^−1^) were purchased from Sigma-Aldrich (Merck KGaA, Darmstadt, Germany). Acetic acid was supplied by Chimopar (CHIMOPAR Trading S.R.L., Bucharest, Romania). Poly(styrene)-b-poly(4-vinylpyridine) (PS_154_-b-P4VP_381_) block copolymer was obtained as previously described [28], in the presence of sec-butyllithium (1.6 M solution in hexane) as initiator, by sequential anionic polymerization in tetrahydrofuran (THF) under a nitrogen atmosphere at −78 °C. The indices (154 and 381) represent the polymerization degree values obtained for PS by Size Exclusion Chromatography (SEC) analysis, obtained using a Shimadzu LC-20AD liquid chromatograph (SHIMADZU, San Jose, CA, USA) equipped with two Varian PL gel 5 μm MIXED-C columns and a refractive index detector Shimadzu RID-10A (SHIMADZU GmbH, Duisburg, Germany) with PS standards, in THF at 25 °C, and for P4VP by ^1^H Nuclear Magnetic Resonance (NMR) spectroscopy, using deuterated chloroform as solvent on a Bruker Avance 400 spectrometer at 400 MHz (Bruker Corporation, Billerica, MA, USA). Sec-butyllithium and tetrahydrofuran were supplied by Sigma-Aldrich (Merck KGaA, Darmstadt, Germany). All chemicals were used without additional purification to synthesize all materials of interest.

### 2.2. Synthesis of TiO_2_ Nanoparticles

**Figure 1:**
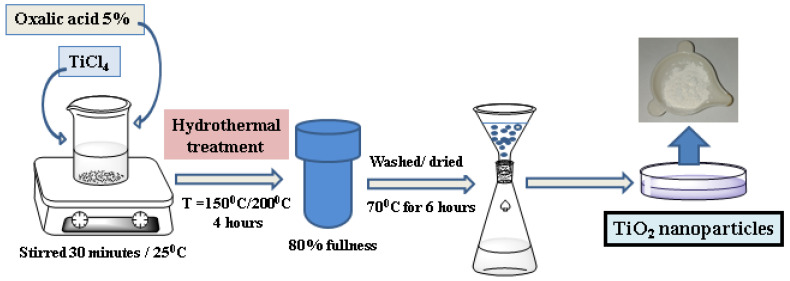
Schematic illustration of the synthesis of TiO_2_ nanoparticles.

The TiO_2_ nanoparticles were synthesized using the classic hydrothermal method: a mixed solution containing 5 mL of TiCl_4_ and 50 mL of oxalic acid 5% was stirred for 30 min to form a homogenous mixture at room temperature (20 ± 1 °C). Afterward, the precipitate was transferred into a Teflon-lined steel autoclave with a fullness degree of 80% at temperatures of 150 °C and 200 °C for 4 h in a calcination oven (Naberthem, Germany). Finally, when the autoclave was cooled in air, the obtained material was filtered using filter paper (Ꝋ125 mm, Macherey-Nagel, Germany), washed with distilled water, and dried in a drying oven (Thermo Scientific Heraeus, type 6060 UT, Hamburg, Germany) at 70 °C for 6 h. The schematic synthesis of the TiO_2_ nanoparticles is illustrated in Figure 1.

### 2.3. Synthesis of TiO_2_ Nanoparticles Polymeric Membrane

**Figure 2:**
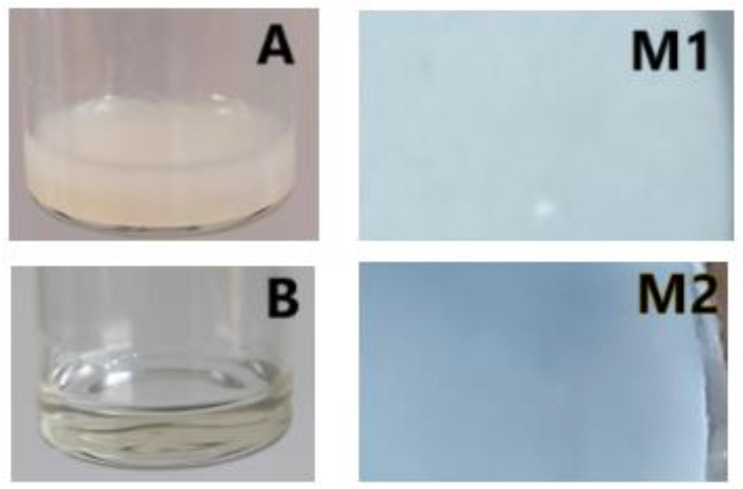
Images of the obtained solutions and the top surface of the obtained materials: polymeric solution with TiO_2_ nanoparticles (**A**), polymeric solution without TiO_2_ nanoparticles (**B**), polymeric membrane with TiO_2_ nanoparticles (CA/PEG/PS_154_-b-P4VP_381_/TiO_2_ membrane (M1)), and polymeric membrane without TiO_2_ nanoparticles (CA/PEG/PS_154_-b-P4VP_381_ membrane (M2)).

The TiO_2_ nanoparticles polymeric membrane was obtained using the wet-phase inversion method at room temperature (22 ± 1 °C) as follows: in a Berzelius beaker, CA (0.3 g) and PEG (2.03 g) were dissolved under magnetic stirring (300 rpm) for 2 h (DLAB MS-H380 Pro, DLAB SCIENTIFIC CO., Beijing, China) in 3 mL of acetic acid (solvent). After that, the poly(styrene)-b-poly(4-vinylpyridine) (PS_154_-b-P4VP_381_) block copolymer (powder) (0.02 g) was added to the mixture obtained. The obtained solution was heated at 100 °C and mixed under continuous magnetic stirring (300 rpm) for 2 h until the complete dissolution of constituents and obtaining of a clear solution. TiO_2_ nanoparticles (0.04 g) were gradually added to the obtained solution under continuous magnetic stirring (300 rpm) and heating for another 1 h, resulting in a homogeneous and viscous solution. The final solution obtained was left to cool at room temperature for 20 min then poured onto a glass plate using a knife. The obtained film was immediately immersed in a glass vessel with distilled water to detach the film as a polymer membrane. To stabilize the structure, the obtained polymeric membrane was kept at room temperature (22 ± 1 °C) for 24 h, finally resulting in a uniform, homogeneous, compact, elastic, and white polymeric membrane (CA/PEG/PS_154_-b-P4VP_381_/TiO_2_ (M1)). To highlight the influence of TiO_2_ nanoparticles on the polymeric membrane matrix, a polymeric membrane without these nanoparticles was prepared (CA/PEG/PS_154_-b-P4VP_381_ membrane (M2)) using the same preparation method. The obtained polymeric solutions with (A) and without (B) TiO_2_ nanoparticles and the top surface of obtained polymeric membranes (M1 and M2) are illustrated in Figure 2.

The thickness of each obtained polymeric membrane was around 0.3 mm. The measurements were performed using a high-precision electronic digital caliper with a digital display (Dasqua 2015-1005-A, Dasqua S.R.L., Cornegliano Laudense, Italy).

**Figure 3:**
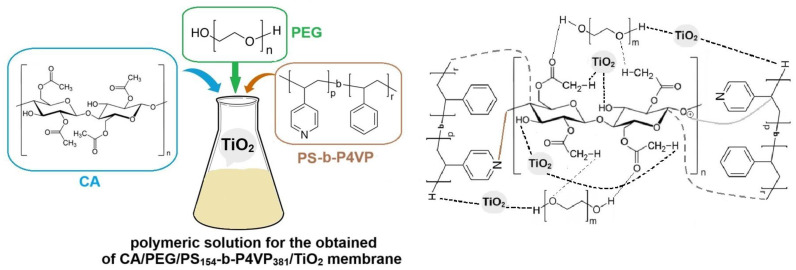
Schematic illustration mechanism between additives and TiO_2_ nanoparticles for the fabrication of the CA/PEG/PS_154_-b-P4VP_381_/TiO_2_ membrane.

Because not all polymers are miscible in contact with solvents, we chose CA and PEG as a matrix for polymeric membrane preparation, which was cross-linked due to the reaction between the acetyl groups in CA, the free hydroxy groups in PEG, and the methyl group in acetic acid. PEG, a hydrophilic aliphatic polymer, could lead to an increased interaction between TiO_2_ nanoparticles and the polymer matrix. During the phase inversion process, the hydrophilic TiO_2_ nanoparticles increase the diffusion velocity of the coagulating bath into the membrane. Also, due to the decrease in the interaction between the polymer and the solvent molecules caused by the steric obstruction instigated by the TiO_2_ nanoparticles, the solvent can disperse very easily from the polymer matrix in the presence of TiO_2_ nanoparticles [5]. The possible mechanism interactions between polymers, copolymers, and TiO_2_ nanoparticles, which may occur during the wet-phase inversion process for the fabrication of the CA/PEG/PS_154_-b-P4VP_381_/TiO_2_ membrane, are schematically illustrated in Figure 3.

The composition and manufacturing process of polymeric membranes does not involve high costs in terms of the necessary raw materials. This feature makes the manufacturing process more accessible and economical, contributing to its widespread implementation in various industrial and commercial contexts for fabrication of the different polymeric membranes. The manufacturing process adopted for obtaining the polymeric membranes is simple, fast, and economically and ecologically sustainable. It does not require the use of expensive polymers and copolymers and does not generate additional waste, thus contributing to reducing the impact on the environment. The process is easy to apply and handle, facilitating the integration of polymeric membranes into various waste-water treatment systems (electrodialysis, ultrafiltration, microfiltration, reverse osmosis, and many more).

### 2.4. Characterization

#### 2.4.1. Morpho-Structural Characterization of the TiO_2_ Nanoparticles

The crystallinity, purity, and phase of the prepared TiO_2_ nanoparticles were measured by X-ray diffraction (XRD, PANalytical X’Pert PRO MPD Diffractometer, Almelo, The Netherlands) with CuKα radiation (λ = 1.54056 Å at 15 mA and 30 kV) in the range of 2θ = 20–80°. The morphology of the materials was examined using scanning electron microscopy (SEM, FEI Inspect S model, Eindhoven, The Netherlands) coupled with the energy dispersive X-ray analysis detector (EDX). To determine the band gap Eg by plotting Kubelka–Munk function against energy (eV), the optical measurements were recorded by UV-Vis analysis using diffuse reflectance mode (PerkinElmer Lambda 950 UV/Vis spectrophotometer, Shelton, CT, USA).

#### 2.4.2. Morpho-Structural Characterization of the Polymeric Membranes

The successfully fabricated polymeric membranes, embedded with and without the obtained TiO_2_ nanoparticles, were ascertained through comprehensive characterization, employing Fourier-Transform Infrared (FTIR) spectroscopy, contact angle measurements, surface free energy (SFE), Scanning Electron Microscopy (SEM), and Energy-Dispersive X-ray Spectroscopy (EDXS).

Fourier-Transform Infrared (FTIR) spectroscopy (Perkin Elmer Spectrum 100 FTIR spectrophotometer, PerkinElmer, Ltd, London, UK) in the Transmittance (T%) mode, in the spectral width ranged between 4000 and 600 cm^−1^ for 16 accumulation scans, and at a spectral resolution of 4 cm^−1^ was used to confirm the interaction between raw materials (polymers and copolymer) and TiO_2_ nanoparticles, and also to identify the functional groups of the fabricated polymeric membranes.

Static contact angle measurements were carried out using a Contact Angle Meter (Kyowa Surface Chemistry Co., Ltd, Tokyo, Japan), KSV instruments CAM 100 equipment (KSV Instruments, Helsinki, Finland). The membrane’s static contact angles were measured at room temperature by placing a 10 µL droplet of distilled water on the surfaces. An average of three measurements ± Standard Deviation, to minimize experimental errors, was reported. The static contact angle registered between each sample membrane (2 cm × 2 cm) and liquids like water, dimethyl sulfoxide (DMSO), and glycerol was measured, and surface free energy (SFE) was calculated.

The surface morphologies of the fabricated polymeric membranes were characterized using Scanning Electron Microscopy (SEM) on an FEI electron microscope (Thermo Fisher, Eindhoven, The Netherlands). Secondary electron imaging was conducted at an accelerating voltage of 30 keV, coupled with energy-dispersive X-ray spectroscopy (EDXS) for elemental analysis. Prior to analysis, the membranes were sectioned into small pieces, sputter-coated with a thin gold layer, and securely mounted in the microscope’s analysis chamber.

#### 2.4.3. Thermogravimetric Analysis (TGA) of the Polymeric Membranes

The TGA measurements of the prepared polymeric membranes were performed using a TGA Q5000 IR system (TA Instruments, New Castle, DE, USA). The analysis was conducted from 30 °C to 700 °C under a nitrogen atmosphere (99.99%, 50 mL/min) with a ramp rate of 10 °C/min in platinum pans (100 μL).

#### 2.4.4. Water Retention and Porosity of the Polymeric Membranes

The water retention capacity and porosity of polymeric membranes were determined using the gravimetric method to check the liquid penetration. The polymeric membranes were cut into small pieces and soaked in distilled water for 48 h. Before being dried, the polymeric membranes were weighed using an analytical balance KERN ADB 100-4 (Kern & Sohn GmbH, Balingen, Germany). Then, the surface moisture of the polymeric membranes was wiped off with filter paper. The polymeric membranes were dried in an oven at 80 °C for 6 h and then were weighed to determine the dry weight of the polymeric membranes.

The degree of water retention capacity (WRC(%)) was determined by using Equation (1) [5,10,29]:

(1) $$WRC\left(\%\right)=\frac{m_{wet}-m_{dry}}{m_{wet}}\times 100$$

The porosity (Ɛ(%)) of polymeric membranes was determined using Equation (2) [29,30,31].

(2) $$Ɛ\left(\%\right)=\frac{m_{wet}-m_{dry}}{S\cdot \rho_{dw}\cdot \sigma }\times 100$$

where m_wet_ and m_dry_ are the weight of wet and dry polymeric membranes (g), respectively, S is the surface of the polymeric membrane (cm^2^), ρ_dw_ is the distilled water density, and σ is the thickness of the polymeric membrane (cm).

## 3. Results and Discussion

### 3.1. Characterization of the TiO_2_ Nanoparticles

**Figure 4:**
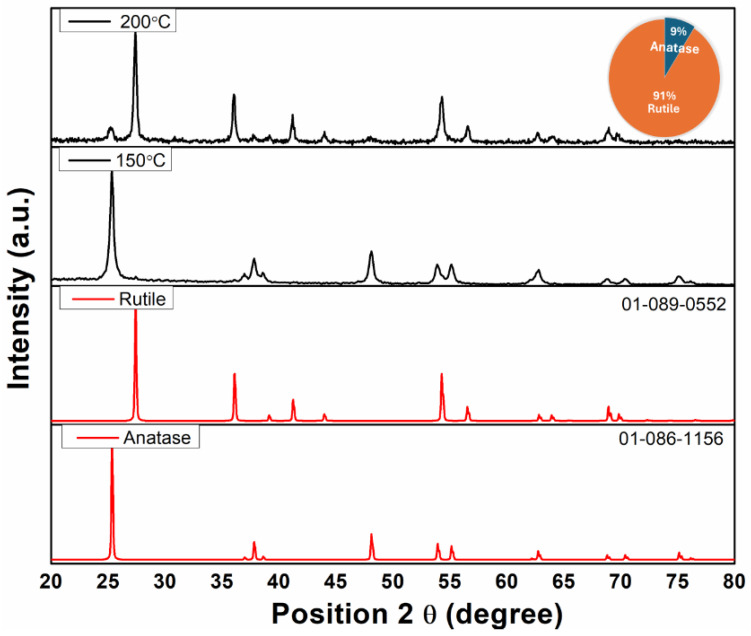
XRD patterns of TiO_2_ nanoparticles synthesized by classic hydrothermal method.

The XRD patterns of TiO_2_ nanoparticles annealed at different temperatures are illustrated in Figure 4.

It is known that annealing improves the crystallization of TiO_2_ powders and accelerates the transformation from the low crystalline phase to the anatase or rutile phase. From Figure 4, it can be seen that at a temperature of 150 °C, the crystalline phase of TiO_2_ nanoparticles reveals an anatase form, corresponding to 2θ: 25.4°, 37.1°, 37.8°, 38.5°, 48.3°, 54.04°, 55.2°, 62.7°, 69°, 70.3°, 75° (JCDS 01-086-1156) [10,32]. The two crystalline phases appear by increasing the temperature to 200 °C, and the rutile phase is predominant. The specific peaks identified for the rutile phase correspond to 2 θ: 27.3°, 36°, 37.8°, 41.2°, 54.3°, 56.6° (JCDS 01-089-0552) [33].

For the fabrication of the polymeric membrane, only pure anatase TiO_2_ nanoparticles were used; these were obtained at a temperature of 150 °C. This was due to a higher anatase phase content of approximately ~99%, which was calculated by fitting the XRD pattern using the X’Pert HighScore Plus Program.

**Figure 5:**
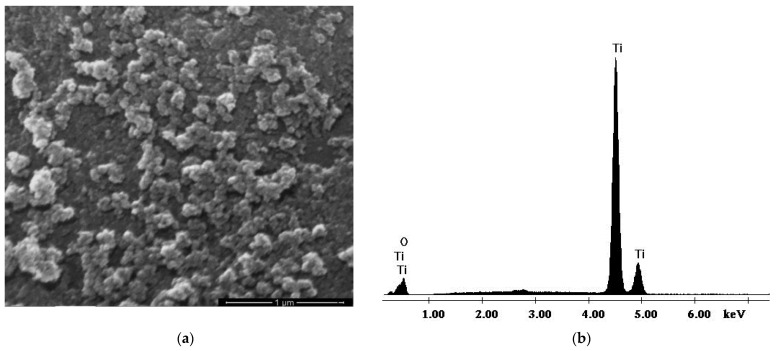
SEM morphology (**a**) and EDX analysis (**b**) for synthesized TiO_2_ nanoparticles.

The morphology of synthesized TiO_2_ nanoparticles was assessed via SEM in conjunction with EDX (Figure 5).

Figure 5a exhibits the SEM image of the synthesized TiO_2_ nanoparticles, which confirms that the nanoparticles are spherical, well-differentiated, and agglomerated in asymmetric conglomerates with clean and smooth surfaces. The size of the nanoparticles is between 10 nm and 20 nm. EDX analysis confirms the purity of the synthesized TiO_2_ nanoparticles, showing only specific peaks for titanium (Ti) and oxygen (O) elements (Figure 5b).

**Figure 6:**
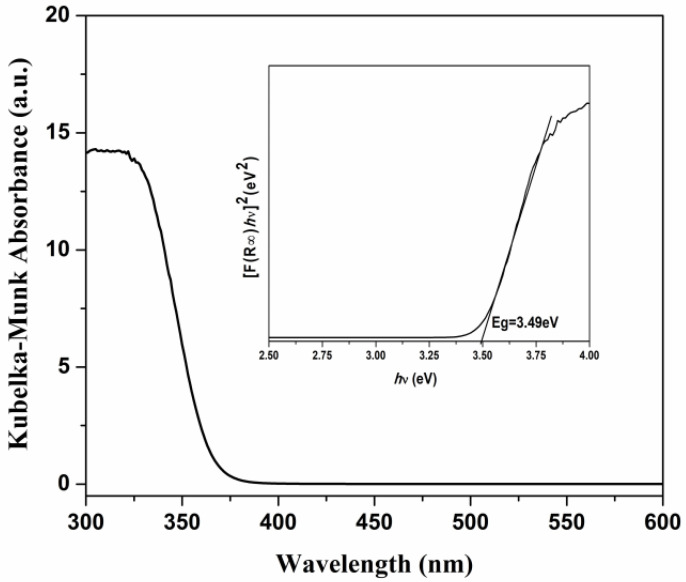
UV-Vis absorption spectra for the TiO_2_ nanoparticles; Inset: Band gap calculation Eg against energy (eV) for the TiO_2_ nanoparticles.

The UV-Vis spectra of the TiO_2_ nanoparticles in the range between 300 nm and 600 nm are presented in Figure 6.

Figure 6 shows that the TiO_2_ nanoparticles absorb only in the UV domain with a wavelength of less than 390 nm. The optical bandgap energy of the TiO_2_ nanoparticles was calculated by the *Tauc* plot using Equations (3) and (4):

(3) $$Eg=\frac{1240}{\gamma }$$

(4) $$\alpha h\nu =A\cdot (h\nu -Eg{)}^{n}$$

where *α*, hν, A, and n denote the absorption coefficient, photon energy, a constant, and an exponent, respectively.

The Eg optical band gap energy is derived from the intersection of the straight line with the hν-axis of the *Tauc* plot [34] (Inset of Figure 6). The estimated band gap of the TiO_2_ nanoparticles is 3.49 eV (Inset of Figure 6), which corresponds to the anatase form of TiO_2_ nanoparticles [35].

### 3.2. Characterization of the Polymeric Membranes

**Figure 7:**
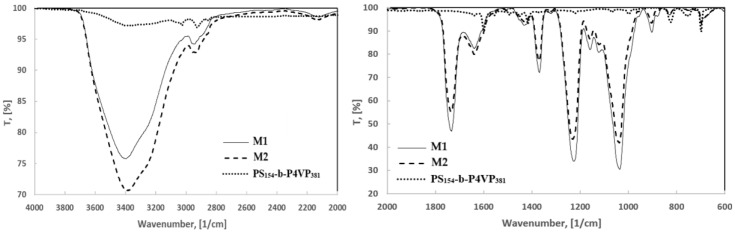
FTIR spectra of CA/PEG/PS_154_-b-P4VP_381_/TiO_2_ membrane (M1), CA/PEG/PS_154_-b-P4VP_381_ membrane (M2), and PS_154_-b-P4VP_381_ copolymer.

The FTIR spectra of the polymeric membranes (CA/PEG/PS_154_-b-P4VP_381_/TiO_2_ membrane (M1), CA/PEG/PS_154_-b-P4VP_381_ membrane (M2)) and copolymer (PS_154_-b-P4VP_381_) are shown in Figure 7.

The FTIR spectrum of pure PS_154_-b-P4VP_381_ (Figure 7) evidenced the main absorption bands [36] as follows: the absorption bands at 3023 cm^−1^ and 2925 cm^−1^ related to the aromatic stretching vibrations of -CH_2_ groups; the band at 1639 cm^−1^ and the sharp absorption band at 1600 cm^−1^ are attributed to C=N vibration in pyridine rings from the P4VP_381_ blocks; an intense peak at 1556 cm^−1^ is attributed to C=C vibration in pyridine rings from the P4VP_381_ blocks; the bands at 1491 cm^−1^ and 1450 cm^−1^ correspond to the phenyl rings of PS_154_ blocks; the band at 1417 cm^−1^ is specific to the C=N stretching vibration mode of aromatic pyridine ring from the P4VP_381_ blocks; and in-plane and out-of-plane C–H bending at 1068 cm^−1^ and ~1001 cm^−1^, respectively, from the pyridine ring.

The FTIR spectra of CA/PEG/PS_154_-b-P4VP_381_/TiO_2_ membrane (M1) and of CA/PEG/PS_154_-b-P4VP_381_ membrane (M2) are indicated in Figure 7. The absorption band corresponding to the stretching vibration of the O-H group was registered at 3390 cm^−1^ in the case of the CA/PEG/PS_154_-b-P4VP_381_/TiO_2_ membrane (M1) and at 3397 cm^−1^ in the case of the CA/PEG/PS_154_-b-P4VP_381_ membrane (M2) (Figure 7) [8]. The appearance of this band at different wavenumbers and a slight increase in this peak intensity in the case of CA/PEG/PS_154_-b-P4VP_381_/TiO_2_ membrane spectrum confirmed the associated water molecules in the presence of TiO_2_ nanoparticles. In all spectra, the absorption band corresponding to the carbonyl of an acetyl group from CA appeared as an intense peak at 1734 cm^−1^, suggesting an O–H· · ·C=O interaction between CA and PEG [8]. Regarding the presence of PS_154_-b-P4VP_381_ in the composition of the CA/PEG/PS_154_-b-P4VP_381_ membrane (M2), the specific absorption bands of the pyridine ring from P4VP_381_ were recorded at 1639 cm^−1^ and 1607 cm^−1^, compared to the intense peak at 1600 cm^−1^ as recorded in the pure block copolymer. However, after adding TiO_2_ nanoparticles, the absorption band at 1607 cm^−1^ was slightly more intense, indicating the interaction of the N atom of pyridine rings from the P4VP_381_ blocks with the Ti ions. The absorption band registered at around 1450 cm^−1^ confirmed the presence of the phenyl rings from the PS_154_ in CA/PEG/PS_154_-b-P4VP_381_ and CA/PEG/PS_154_-b-P4VP_381_/TiO_2_ membranes. In the case of CA/PEG/PS_154_-b-P4VP_381_ membrane (M1), the absorption band specific to the deformation vibration of C-H in CA and PEG appeared at 1433 cm^−1^. Still, when TiO_2_ nanoparticles were added, the band’s intensity decreased. The C=N stretching vibration of the pyridine ring from P4VP_381_ blocks registered in block copolymer at 1417 cm^−1^ for the CA/PEG/PS_154_-b-P4VP_381_/TiO_2_ membrane (M1) was shifted to 1423 cm^−1^ for the CA/PEG/PS_154_-b-P4VP_381_ membrane (M2). However, the peak area at 1423 cm^−1^ increased when both PS_154_-b-P4VP_381_ and TiO_2_ nanoparticles were added into the polymeric membrane composition, indicating that a supramolecular structure was obtained, in which the nitrogen atoms of the pyridine ring favored the coordination with Ti ions. The absorption band specific to the stretching vibration of a glycosidic ring from CA (ν_C-O-C_) appeared in all membranes as an intense peak at around 1036 cm^−1^. In the case of CA/PEG/PS_154_-b-P4VP_381_/TiO_2_ membrane, the presence of Ti-O-Ti bonds was identified at ~678 cm^−1^ [8]. These results confirm the successful synthesis of the CA/PEG/PS_154_-b-P4VP_381_/TiO_2_ membrane.

The contact angle measurements and the stability of a material’s hydrophilic/hydrophobic character are important in establishing its performance in various membrane processes for waste-water treatment. The direct contact method between water, DMSO, or glycerol drops and membranes was used, and the values of the contact angles registered showed an important change in the wetting properties of the membranes (Table 1).

The differences in contact angle values obtained for polymeric membranes could be attributed to the hydrophilic/hydrophobic character of PS_154_-b-P4VP_381_ chain segments and CA and PEG additives in the polymeric membranes. A higher water contact angle value was registered on the CA/PEG/PS_154_-b-P4VP_381_ membrane (M2) (75.62 ± 1.13°) compared to the CA/PEG/PS_154_-b-P4VP_381_/TiO_2_ membrane (M1) (15.57 ± 0.45°). Initially, PS_154_-b-P4VP_381_ came with the hydrophilic (P4VP_381_) and the hydrophobic (PS_154_) parts [37,38], but when both PS_154_-b-P4VP_381_ and TiO_2_ nanoparticles were included, an abrupt decrease in the water contact angle value from 75.62 ± 1.13° (M2) to 15.57 ± 0.45° (M1) was noticed, showing a pronounced hydrophilic character of the CA/PEG/PS_154_-b-P4VP_381_/TiO_2_ membrane (M1). This behavior can be explained by the coordination of nitrogen atoms of the pyridine ring from the P4VP_381_ blocks with Ti ions. Ahmad et al. [10] obtained a polyurethane/cellulose acetate membrane modified with functionalized TiO_2_ nanoparticles. The obtained results indicated that the contact angle values decrease with the increasing concentration of TiO_2_ nanoparticles in the polymeric matrix. The higher contact angle value of 79° ± 0.1 was obtained for the membrane with 0.25% of TiO_2_, and the lower contact angle value of 55° ± 0.1 was obtained for membrane with 1.25% of TiO_2_. Pereira et al. [29] obtained membranes containing polysulfone and sulfated TiO_2_ nanoparticles. They related that the contact angle was at least 60° for the membrane, which contains a concentration of sulfated TiO_2_ nanoparticles of 1.0 wt%. Goyat et al. [31] related that the value of the contact angle was 42.5° for the fabricated membrane containing graphene oxide-TiO_2_ blended with polyether sulfone. Liang et al. [39] reported that the water contact angle for the polyvinyl alcohol@5%TiO_2_/carboxyl-polyether sulfone membrane was 43.1°.

To evaluate the SFE of the membranes, the contact angle against three liquids, the Owens–Wendt’s theory, and the Fawkes method [40,41] were used. Values of the dispersive $\gamma_{L}^{d}$ and polar $\gamma_{L}^{p}$ components of the surface tension were used as follows: water ($\gamma_{L}^{d}\;$ = 21.8 mN m^−1^ and $\gamma_{L}^{p}\;$ = 51 mN m^−1^); DMSO ($\gamma_{L}^{d}\;$ = 36 mN m^−1^ and $\gamma_{L}^{p}\;$ = 8 mN m^−1^); glycerol ($\gamma_{L}^{d}$ = 37 mN m^−1^ and $\gamma_{L}^{p\;}$ = 26.4 mN m^−1^). The obtained total surface free energy $\gamma_{S}$ of the membranes and dispersive $\gamma_{S}^{d}$ and polar $\gamma_{S}^{p}$ components of the solid surface energy are summarized in Table 2.

Analyzing Table 1 and Table 2, the decrease in water contact angle value from 75.62 ± 1.13° (M2) to 15.57 ± 0.45° (M1) (Table 1) and the increase in $\gamma_{S}$ from 30.0623 mN m^−1^ (M2) to 74.7370 mN m^−1^ (M1) can be observed. The incorporation of TiO_2_ nanoparticles determined a significant increase in the hydrophilicity of the polymeric membrane (M1), and the increase in polar components led to an increase in energy values. This may be due to the high surface energy of the metal [42].

**Figure 8:**
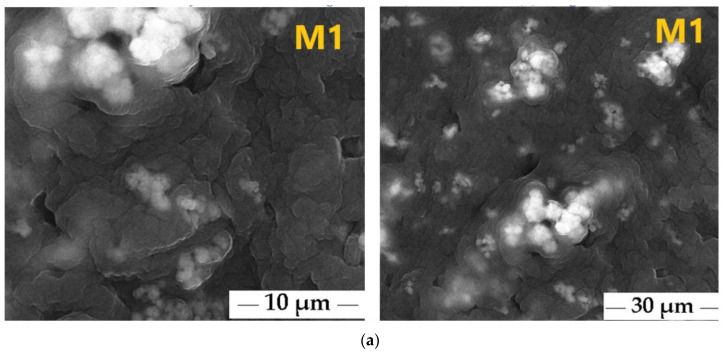

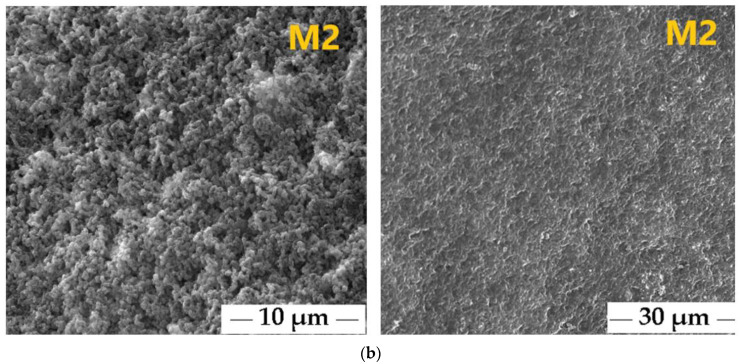
SEM images of fabricated polymeric membranes: (**a**) CA/PEG/PS_154_-b-P4VP_381_/TiO_2_ membrane (M1) at magnifications of 10,000× and 5000×; (**b**) CA/PEG/PS_154_-b-P4VP_381_ membrane (M2) at magnifications of 10,000× and 5000×.

The surface morphologies of the fabricated polymeric membranes, CA/PEG/PS_154_-b-P4VP_381_/TiO_2_ membrane (M1) and CA/PEG/PS_154_-b-P4VP_381_ membrane (M2), were examined using scanning electron microscopy (SEM) at magnifications of 10,000× and 5000×, as shown in Figure 8.

The samples were further characterized using SEM. The pore diameters of the CA/PEG/PS_154_-b-P4VP_381_/TiO_2_ membrane (M1) and the CA/PEG/PS_154_-b-P4VP_381_ membrane (M2) are shown in Figure 8. The M1 membrane exhibits smaller pores, with diameters ranging from 50 nm to 70 nm (Figure 8a), compared to the M2 membrane, which shows pore diameters between 90 nm and 110 nm (Figure 8b). Figure 8 demonstrates the homogeneous mixing of CA with PEG in the polymeric membranes matrix.

The SEM images of the CA/PEG/PS_154_-b-P4VP_381_ membrane (M2) (Figure 8b) reveal a rough surface, a dense structure featuring macro-voids, and a high density of pores. In contrast, incorporating the TiO_2_ nanoparticles in the CA/PEG/PS_154_-b-P4VP_381_/TiO_2_ membrane (M1) significantly modified its surface morphology, as depicted in Figure 8a. The SEM analysis indicates that the TiO_2_ nanoparticles were well dispersed and embedded within the polymeric matrix of the M1 membrane.

The spherical morphology of the M1 membrane, characterized by a compact, smooth, and uniform surface with reduced pore size, suggests strong interactions between the polymeric additives and TiO_2_ nanoparticles during the wet-phase inversion process. However, Figure 8a also shows that the TiO_2_ nanoparticles tend to agglomerate within the polymeric matrix. According to the literature, such agglomeration of the TiO_2_ nanoparticles has been reported to enhance membrane efficiency and permselectivity [8,24]. Wibowo et al. [8] indicated that the pore size of the membrane synthesized based on cellulose acetate/PEG/chitosan and TiO_2_ nanoparticles was between 10 nm and 1000 nm. Ahmad [10] reported on fabricated membranes containing polyurethane-cellulose acetate and different concentrations of TiO_2_ nanoparticles. After investigation of the surface morphologies of the membranes, it was observed that the membrane with a lower concentration of TiO_2_ nanoparticles (0.5%) showed an average pore size between 1500 nm and 2500 nm, and the membrane with higher concentration of TiO_2_ nanoparticles (1.5%) showed an average pore size between 300 nm and 500 nm. Pereira et al. [29] reported that the membranes’ morphology changed after adding sulfated-TiO_2_ nanoparticles (s-TiO_2_) to the polymeric matrix of the polysulfone membrane. The investigations indicated that the membrane containing a higher concentration of s-TiO_2_ (2.0 wt%) formed larger aggregates and, therefore, blocked the pores of the polysulfone membrane.

**Figure 9:**
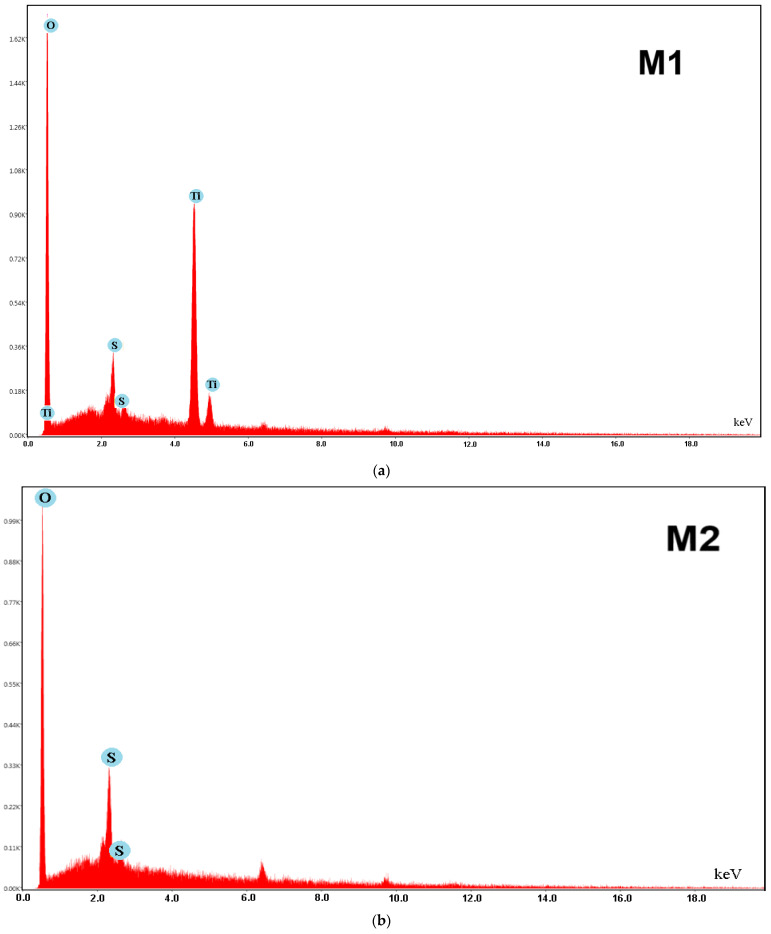
EDXS spectra: (**a**) CA/PEG/PS_154_-b-P4VP_381_/TiO_2_ membrane (M1); (**b**) CA/PEG/PS_154_-b-P4VP_381_ membrane (M2).

The EDXS spectra (Figure 9a,b) illustrate the elemental composition of the polymeric membranes, confirming the presence of sulfur (S) and oxygen (O) elements, as reported in previous studies [8,43]. Additionally, Figure 9a highlights the presence of titanium (Ti) in the CA/PEG/PS_154_-b-P4VP_381_/TiO_2_ membrane (M1), confirming the successful incorporation of the TiO_2_ nanoparticles. These findings are consistent with the results obtained from the FTIR analysis, further validating the synthesis process.

Table 3 shows the water retention and the porosity values of the fabricated polymeric membranes (CA/PEG/PS_154_-b-P4VP_381_/TiO_2_ membrane (M1) and CA/PEG/PS_154_-b-P4VP_381_ membrane (M2)).

The higher water retention and porosity values obtained for the CA/PEG/PS_154_-b-P4VP_381_/TiO_2_ membrane confirm the impact and the role of TiO_2_ in the polymeric membrane matrix, as well as in enhancing pore formation. The obtained values confirmed that the CA/PEG/PS_154_-b-P4VP_381_/TiO_2_ membrane is more hydrophilic and porous than the CA/PEG/PS_154_-b-P4VP_381_ membrane. The hydrophilicity of the CA/PEG/PS_154_-b-P4VP_381_/TiO_2_ membrane will promote the diffusion of water through the membrane [29]. The improved hydrophilicity, high porosity, and high water retention make the CA/PEG/PS_154_-b-P4VP_381_/TiO_2_ membrane highly efficient in water purification and waste-water treatment through membrane processes. Ahmad et al. [10] reported that the water retention of polyurethane/cellulose acetate and TiO_2_-modified membranes was influenced by the amount of TiO_2_ nanoparticles. The lowest value of water retention (~43%) was obtained for the membrane containing 0.75% TiO_2_, and the highest water retention value (~92%) was obtained for the membrane containing 1.25% TiO_2_. Pereira et al. [29] indicated that the water retention and the porosity of the membranes containing polysulfone/sulfated TiO_2_ nanoparticles (s-TiO_2_) depend on the concentration of s-TiO_2_ (0.05 wt%–2.0 wt%, weight percent). The lowest water retention (29.2%) and porosity (15.3%) were obtained for the membrane containing 0.05 wt% s-TiO_2_, and the highest water retention (47.2%) and porosity (31.3%) were obtained for the membrane containing 2.0 wt% s-TiO_2_.

**Figure 10:**
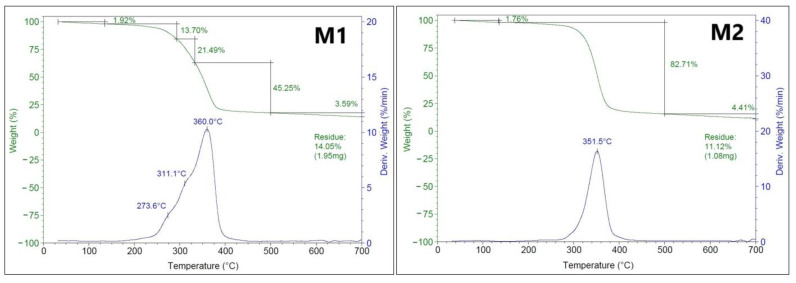
TGA and the corresponding temperature derivative curves of the CA/PEG/PS_154_-b-P4VP_381_/TiO_2_ membrane (M1) and the CA/PEG/PS_154_-b-P4VP_381_ membrane (M2).

The thermal stability of the obtained polymeric membranes was studied using the TGA and the corresponding temperature derivative curves (Figure 10).

The TGA curves indicated that the prepared polymeric membranes (CA/PEG/PS_154_-b-P4VP_381_/TiO_2_ membrane (M1) and CA/PEG/PS_154_-b-P4VP_381_ membrane (M2)) have different thermal loss profiles. In all curves, the weight loss between 30 °C and 135 °C, for the M1 membrane of 1.92% and the M2 membrane of 1.76%, was attributed to the dehydration and evaporation of bounding water [10,43]. The weight loss between 135 °C to 500 °C, which for the M1 membrane was 45.25% and for the M2 membrane was 82.71%, can be attributed to the deacetylation process and disintegration of acetyl groups and hydroxy groups from polymeric matrix chain (CA/PEG) [10]. The final weight loss between 500 °C to 700 °C, which for the M1 membrane was 3.59% and the M2 membrane was 4.41%, was mainly due to the carbonization of the decomposed product to ash. The small difference in residual polymer mass can be attributed to the presence of TiO_2_ nanoparticles in the polymeric matrix: for the CA/PEG/PS_154_-b-P4VP_381_/TiO_2_ membrane (M1) it was 1.95 mg, and for the CA/PEG/PS_154_-b-P4VP_381_ membrane (M2) it was 1.08 mg. In the case of the CA/PEG/PS_154_-b-P4VP_381_/TiO_2_ membrane (M1), the weight losses between 135 °C and ~295 °C and between ~295 °C and ~335 °C can be attributed to the interaction between polymers (CA and PEG), the copolymer (PS_154_-b-P4VP_381_), and fabricated TiO_2_ nanoparticles in the polymeric membrane matrix. The TGA curves of the prepared polymeric membranes show excellent thermal stability; the maximum decomposition temperature values were 360 °C for the CA/PEG/PS_154_-b-P4VP_381_/TiO_2_ membrane (M1) and 351.5 °C for the CA/PEG/PS_154_-b-P4VP_381_ membrane (M2). The TGA curve indicated that the incorporation of TiO_2_ nanoparticles into the polymeric membrane matrix improved the thermal stability and properties of the prepared CA/PEG/PS_154_-b-P4VP_381_/TiO_2_ membrane. The results indicated that the prepared polymeric membranes are thermally stable compared to other fabricated membranes containing TiO_2_ nanoparticles. Ahmad et al. [10] prepared membranes containing polyurethane/cellulose acetate and functionalized with different concentrations of TiO_2_ nanoparticles (between 0.5% and 1.50%). They reported that only the membrane with the highest concentration of TiO_2_ nanoparticles (1.50% weight percent) had a higher thermal stability (305 °C). Aparicio et al. [22] reported that the decomposition of the obtained polymer membranes based on the polyvinyl alcohol/TiO_2_ nanoparticles occurred above 250 °C.

The results indicate that the CA/PEG/PS_154_-b-P4VP_381_/TiO_2_ membrane obtained by the wet-inversion method has many advantages and properties compared to other membranes reported in other studies [10,22,29,31], such as: was obtained from the additives and raw materials that are accessible, abundant, an non-toxic, low costs, environmentally friendly, and exhibits high chemical, structural, and thermal stability.

## 4. Conclusions

In our study, we successfully synthesized TiO_2_ nanoparticles by the hydrothermal method and also successfully fabricated a novel polymeric membrane from macromolecular compounds with functional groups and TiO_2_ nanoparticles by the wet-phase inversion method. The SEM image indicated that the size of TiO_2_ nanoparticles was between 10 and 20 nm. The EDX analysis confirms the purity of the fabricated TiO_2_ nanoparticles. The obtained CA/PEG/PS_154_-b-P4VP_381_/TiO_2_ and CA/PEG/PS_154_-b-P4VP_381_ membranes were characterized by FTIR, SEM, EDXS, and TGA. The obtained results revealed that adding TiO_2_ nanoparticles in the polymeric matrix improves the chemical structure, surface morphology, pore size and formation, and the surface properties of the polymeric membrane. The water contact angle measurement indicated that the hydrophilic property of the CA/PEG/PS_154_-b-P4VP_381_ membrane was significantly improved by the addition of fabricated TiO_2_ nanoparticles. The values obtained for the water contact angle of 15.57 ± 0.45°, the water retention of 81.60%, and the porosity of 87.35% for the CA/PEG/PS_154_-b-P4VP_381_/TiO_2_ membrane confirmed the favorable effect of fabricated TiO_2_ nanoparticles added to the polymeric membrane matrix. The results demonstrated that the CA/PEG/PS_154_-b-P4VP_381_/TiO_2_ membrane obtained with environmentally safe materials and with remarkable properties (hydrophilic character, higher water retention, higher porosity, physicochemical stability, and excellent thermal stability) is suitable for a wide variety of applications, specifically in the membrane processes for waste-water treatment (electrodialysis, nanofiltration, ultrafiltration, reverse osmosis, and many more).

## Figures and Tables

**Table 1:** Contact angle values of membranes.

Liquid	Tyle of Polymeric Membrane	
	CA/PEG/PS_154_-b-P4VP_381_/TiO_2_ (M1)	CA/PEG/PS_154_-b-P4VP_381_ (M2)
Water	15.57 ± 0.45°	75.62 ± 1.13°
DMSO	18.86 ± 0.24°	42.73 ± 0.40°
Glycerol	89.13 ± 0.35°	75.94 ± 0.62°

**Table 2:** SFE and its polar and dispersive components for polymeric membranes.

Tyle of Polymeric Membrane	$\gamma_{S}^{P}$ mN m^−1^	$\gamma_{S}^{d}$ mN m^−1^	$\gamma_{S}$ mN m^−1^
CA/PEG/PS_154_-b-P4VP_381_/TiO_2_ (M1)	73.4998	1.2372	74.7370
CA/PEG/PS_154_-b-P4VP_381_ (M2)	11.4258	18.6365	30.0623

**Table 3:** Water retention (WRC (%)) and porosity (Ɛ (%)) values of polymeric membranes.

Tyle of Polymeric Membrane	WRC (%)	Ɛ (%)
CA/PEG/PS_154_-b-P4VP_381_/TiO_2_ (M1)	81.60	87.35
CA/PEG/PS_154_-b-P4VP_381_ (M2)	63.98	64.38

## Data Availability

The original contributions presented in this study are included in the article. Further inquiries can be directed to the corresponding author.
